# Survey on outcomes of emergency standing caesarean section in equids

**DOI:** 10.3389/fvets.2025.1548978

**Published:** 2025-04-24

**Authors:** Geoffroy de la Rebière de Pouyade, Maureen Binard, Stéfan Deleuze, Jérôme Ponthier

**Affiliations:** ^1^Clinical Department of Equids, Surgery and Orthopedics, Faculty of Veterinary Medicine University of Liège, Liège, Belgium; ^2^FARAH Comparative Veterinary Medicine, University of Liège, Liège, Belgium; ^3^Clinical Department of Equids, Theriogenology, Faculty of Veterinary Medicine University of Liège, Liège, Belgium

**Keywords:** horse, equine, standing, caesarean, C-section, prognosis, complication

## Abstract

**Introduction:**

Standing caesarian section (C-section) in mares is poorly documented in the literature. It is reportedly straightforward to perform for veterinarians experienced in bovine practice and is more accessible and less expensive than recumbent techniques. The study aimed to create a survey to report the outcomes of in field C-section through a flank laparotomy with the mare standing under sedation.

**Methods:**

A survey was developed for field veterinarians practicing standing C-sections. Data were collected regarding the veterinarian’s experience and practice type, details about the mare and the reasons for performing the standing C-section in the field, the mare’s condition during the procedure, as well as information on asepsis protocols, anesthesia, surgical techniques, medications used, and the follow-up of complications, including the survival outcomes of both the mare and the foal. Complication and survival rates were then used to determine potential risk factors. Fisher’s exact tests were used, and significance was set at *p* < 0.05.

**Results:**

35 questionnaires, each addressing one C-section case, completed by a total of 19 veterinarians were considered. The most important factor for performing the surgery was the economic concern. The main postoperative complication of the surgery was infection/dehiscence of the wound (12/34). The mare survival rate at 1 year was 74% (25/34). The foal survival rate at 6 months was 20% (7/35). More live foals were delivered when dystocia lasted less than 2 h.

**Conclusion:**

This survey shows that performing a standing C-section in the field might be a viable technique, both economically and in terms of mare’s and foal’s survival.

## 1 Introduction

Caesarean section (C-section) is a surgical procedure by which one or more fetuses are delivered through an incision in the maternal abdominal wall. Elective and emergency C-sections have long been performed in the equine species (1). Elective C-section is indicated for mares suffering from conditions affecting the pelvic canal or the abdominal wall, whereas the main indication for emergency C-section is dystocia, but it can occasionally be performed during surgery for colic or uterine torsion close to term (2–4).

Considering all C-section categories, retained placenta seems to be the most common complication occurring in 32 to 75% of cases (1, 2, 5–7), but it can reach 100% of elective cases in some studies (2, 7, 8).

The mare survival rate for a C-section in recumbency (whether or not under general anesthesia), excluding cases of colic, is considered good to excellent, especially in cases of elective C-section, ranging from 81 to 100% (1, 2, 6–12). As observed in the mare, the foal survival rate at discharge for elective C-sections is better than in emergency cases and may be as high as 78 to 100%. Including emergency C-section performed for dystocia or concurrent maternal disease, the foal survival rate at discharge ranges from 11 to 35% and would be more significantly influenced by the duration of the dystocia than the method of resolution (1, 2, 7, 9, 11, 12).

Rapid transfer to the clinic for a C-section is not always possible or general anesthesia may represent an unacceptable risk. Standing C-section is reportedly straightforward to perform, especially for skilled veterinarians in bovine practice, more accessible, and less expensive than the recumbent techniques in a hospital setting (13, 14). Consequently, in-field standing C-section could be a possible alternative, but to the best of our knowledge, this practice remains rarely performed and poorly reported in the literature (13–15). The primary objective of this survey was to evaluate the outcomes of the standing C-section performed by veterinarians in the field, in terms of survival and complication rates both for the mare and the foal. Our secondary objective was to identify the reasons for performing the in-field surgery and the potential risk factors influencing in-field standing C-section survival or complication rates such as wound infection and/or dehiscence.

## 2 Materials and methods

Field veterinarians were invited to answer a survey to report their experiences and results with standing C-sections, with the stated aim of offering a more objective evaluation of this practice. The survey developed included 68 questions (Supplementary Annex 1) and was divided into six parts. The first part focused on the experience of the field veterinarian and the type of practice. The objective of the second part was to obtain information about the mare and the reasons for performing the standing C-section in the field. The third part was designed to describe the condition of the mare during the C-section. The fourth part collected information on the various protocols of asepsis, local anesthesia and sedation, surgery, and medication used. In the fifth part, the practitioners were asked about the complications and the survival rates. Finally, the last part monitored the potential impact of C-sections on future fertility. For some questions, multiple responses were allowed. When non-anticipated responses to questions were possible, a box “other” was left for open responses. The veterinarians were asked to complete one questionnaire per case to avoid any data confusion. Given the supposed rarity of this surgery and the details of some questions, partial completion of the questionnaire was accepted. No time constraint was imposed to report cases.

The questionnaire (Supplementary Annex 1) was transcribed on Google Forms. The questionnaire was distributed via multiple social networks veterinary groups as well as by word-of-mouth. It was also published on a website dedicated to the veterinary profession accessible to practitioners (https://www.vetofocus.com) and an email was sent to all subscribers. Responses received between March 31st and July 1st, 2022, were considered. Categories for the type of practice, for experience in bovine c-sections, for the duration of dystocia, and the duration of the surgery were defined in the survey. For further analysis, the number of years (y) of experience of the practitioner was converted into categorical data (<6y; 6-10y; 11-20y; >20y). The same was performed for the age of the mares (<6y; 6-10y; 11-16y; >16y). Criteria for diagnosing complications were left to the practitioners’ discretion.

Potential factors that could influence both complication rates for foals and mares and their survival were: veterinarian type of practice and experience (in years or in the number of performed bovine C-sections), mare’s breed and age, duration of the labor at the beginning of the C-section (duration of dystocia) and duration of the surgery. Intra- and postoperative complications were also analyzed as risk factors for the foals and mares’ survival.

These data in addition to data collected about the C-section procedure, from the preparation of the patient and the practitioner to closure and protection of the wound, were also analyzed as potential risk factors for the specific complication of infection-dehiscence of the surgical wound. For this analysis, only the type of local or locoregional anesthesia of the flank was investigated in the anesthetic procedure.

Statistical comparisons were performed using Medcalc®. Fisher’s exact tests were performed due to their robustness for small sample sizes and low-frequency counts. Statistical significance was set at *p* < 0.05.

## 3 Results

Thirty-seven questionnaires were received and two were discarded: one was reporting multiple cases without the possibility of individualizing each case for further analysis, and in the other one, the mare was already down at the veterinarian’s arrival. Finally, 35 questionnaires completed by 19 veterinarians were considered for analysis. Because of insufficient collected data, the potential impact of C-sections on future fertility was not further examined. The number of responses per item in the questionnaire is given in Supplementary Annex 1.

Table 1 summarizes data about the originating country, the global experience in years of practice and the number of bovine standing flank c-sections previously performed according to the type of practice of the veterinarians.

**Table 1:** The originating country, the global experience in years (y), and the experience for standing flank c-sections in the cow of the veterinarians according to their type of practice.

Type of practice	Country					Years of practice experience					Number of C-sections performed in the cow			
	France	Belgium	Algeria	Portugal	Total	< 6 y	6 to 10 y	11 to 20 y	>20 y	Total	< 50	50–100	>100	Total
Equine	2			1	3 (16%)		2	1		3	3			3
Rural	1	2			3 (16%)			2	1	3			3	3
Mixed (Equine - pets)	2				2 (10.5%)	1	1			2	2			2
Mixed (Equine - rural)	4	4	1		9 (47%)	1	3	2	3	9	1	1	7	9
Mixed (Equine - pets-rural)		2			2 (10.5%)	2				2			2	2
Total	9 (47%)	8 (43%)	1 (5%)	1 (5%)	19 (100%)	4 (21%)	6 (32%)	5 (26%)	4 (21%)	19	6 (32%)	1 (5%)	12 (63%)	19

Among the 35 mares, 40% (14/35) were draft horses, 20% (7/35) were warmbloods, 17% (6/35) were Shetlands/Miniature ponies, 9% (3/35) were Standardbred, and 14% (5/35) were categorized as ‘other’ breeds. The distributions of age and parity of the treated mares are summarized in Table 2. Only two among the 17 multiparous mares had experienced complications during the previous foaling. For three mares, the number of previous gestations was unknown.

**Table 2:** Age and parity of the mares.

	< 6 years	6–10 years	11–16 years	>16 years	Unknown	Total
Number of mares	8 (22%)	11 (31%)	10 (29%)	3 (9%)	3 (9%)	35
Number of primiparous	7	4	5	0	2	18 (51%)
Number of multiparous	1	7	5	3	1	17 (49%)

Economic concern was the most frequently reported reason for performing in-field C-sections (83%, 29/35). Survival of the mare was a priority more frequently cited (66%, 23/35) than the foal survival (9%, 3/35). The distance from a referring center was reported in 14% (5/35). In 11% (4/35) of cases, the mare was judged unfit for transport.

The duration of dystocia before the start of the C-section was between 30 and 60 min for 6% (2/35) of the mares, and between 1 and 2 h for 29% (10/35) of the mares. Sixty-six percent (23/35) of cases had dystocia lasting longer than 2 h.

Before surgery, 80% of horses were shaved (28/35), 14% (5/35) were clipped and 6% (2/35) were both clipped and shaved. Asepsis of the surgical site was then carried out with chlorhexidine preparations on 80% (28/35) of mares and with povidone-iodine preparations on the remaining mares. Forty-three percent of the cases (15/35) were then rinsed with alcohol, 23% (8/35) with water, 17% (6/35) with chlorhexidine, 9% (3/35) with water and chlorhexidine, 6% (2/35) with water and alcohol, and 3% (1/35) were not rinsed off.

Surgeons used chlorhexidine preparations for hand asepsis in most cases (83%; 29/35). Povidone-iodine preparations were preferred in 11% (4/35) of cases. One of the veterinarians used a classic soap and another one only used hydro-alcoholic gel. Rinsing was performed with alcohol (37%; 13/35), water (29%; 10/35), chlorhexidine solution (17%; 6/35), or both water and chlorhexidine solution (9%; 3/35). Two practitioners (6%) did not rinse their hands, including the one preparing his hands using the hydro-alcoholic gel. The veterinarian who used the classic soap also rinsed his hands with hydro-alcoholic gel (3%).

All but one veterinarian having performed multiple cases used the same personal protective equipment to perform the surgery. The most frequent combination was wearing a non-sterile gown with sterile gloves (6 veterinarians) or non-sterile gloves (4 veterinarians). The two veterinarians performing the surgery with a sterile gown and sterile gloves also wore a mask. Two additional veterinarians used a sterile gown with either non-sterile gloves or no gloves. Two wore only sterile gloves, one only non-sterile gloves and one only a non-sterile gown. Two veterinarians wore neither a gown and gloves. Analgesia and sedation were mainly achieved with a combination of an alpha2-agonist and an opioid (79%, 27/34). A local or loco-regional anesthesia was performed on all reported cases. Epidural was, however, only performed in 26% (9/34) of cases and it was always associated with local analgesia. Details about the various local, locoregional and sedation protocols are summarized in Table 3.

**Table 3:** Local anesthesia and sedation protocols (*n* = 34).

Type of local anesthesia	Alpha 2 adrenergic agonist				No alpha 2 adrenergic agonist			Total
	Morphine	Butorphanol	Butorphanol + Acepromazine	No opioïds	Morphine + Butorphanol	Butorphanol	No opioïds	
Linear block	2	11	1	1	1	1	1	18 (53%)
Inverted L block		4		1			1	6 (18%)
Epidural + Linear block		8						8 (23%)
Epidural + inverted L block		1						1 (3%)
Paravertebral block				1				1 (3%)
Total	2 (6%)	24 (70%)	1 (3%)	3 (9%)	1 (3%)	1 (3%)	2 (6%)	34 (100%)

Most surgeries (57%; 20/35) were performed through the left flank. The placenta was reported to be removed before closing the uterus in 47% (16/34) of cases. When the placenta was left in place, it was separated from the wound margins in 83% (15/18) of cases, and oxytocin was administered postoperatively in 78% of those cases. When suturing the uterus, a hemostatic suture was applied in 46% (16/35) of cases (not including a mare suffering from uterine hemorrhage), and a two-layer closure, including at least one inverting suture, was performed in 94% (33/35) of mares. All practitioners used absorbable suture material, and 82% (27/33) used monofilament sutures.

For muscle closure, 58% (18/31) of cases were sutured in 2 planes, 39% (12/31) in 3 planes, and in one case all muscle layers were sutured together (3%). When the muscle wall was closed in two planes, the transverse and internal oblique muscles were taken together in 89% (16/18) of cases. A simple continuous suture pattern was performed on 89% (31/35) of the mares. The others were closed using an interrupted suture pattern. Sixty-nine percent of veterinarians (22/32) used a multifilament material. In all cases, an absorbable suture material was used. For skin closure, 80% (28/35) of the mare were sutured with an interlocking continuous pattern, 6% (2/35) with a simple continuous pattern, and 14% (5/35) with an interrupted suture pattern. In 74% of cases (25/34), non-absorbable material sutures were used to suture the skin, and multifilament material was preferred in 69% (22/32) of cases.

C-sections were performed in 45 to 90 min in 71% (25/35) of cases. The surgeries were performed in less than 45 min in 9% (3/35) of cases and more than 90 min in 20% (7/35) of the mares.

At the end of surgery, the surgical wound was only prophylactically treated with an antiseptic spray in 53% of cases (18/34). In 38% of cases (13/34), the wound was left uncovered and exposed. In 6% of cases (2/34), the wound was sprayed with an antiseptic/antibiotic and covered with a dressing. In only one case, a bandage was applied (3%). All the mares received antibiotics and all but one (97%; 34/35) a nonsteroidal anti-inflammatory drug. Seventeen mares (49%) received a combination of penicillin/aminoglycoside, nine penicillin only (26%), five cephalosporins (14%), three sulfonamides (9%), and one fluoroquinolone (3%). The median duration of administration (n = 32) was 5 days (percentiles 25–75: 5–7.5).

Fourteen percent of the mares (5/35) spontaneously went down during the surgery. Twenty-nine percent of the mares (10/35) developed complications during surgery. Abdominal contamination by fetal fluid was observed in 14% (5/35) of the mares. One of the mares, which went down during surgery, contaminated her surgical wound with gravel (3%). Difficulties for foal extraction due to the size and/or rigidity were reported in 3 cases (9%), two in draft horses and one in a Shetland. One mare experienced fatal uterine hemorrhage after uterine torsion was resolved (3%), while another developed hemorrhage in the abdominal muscles (3%). Veterinarians having a mixed small animal / equine practice encountered more intraoperative complications (100%; 3/3) than veterinarians having a pure rural practice (0%; 0/4; *p* = 0.028) or having a mixed equine / rural practice (23%; 5/22; *p* = 0.024). Veterinarians with an experience of more than 100 bovine C-sections also encountered fewer intraoperative complications (15%; 4/26) than those who experienced less than 50 bovine c-sections (62%, 5/8; *p* = 0.0174). Performing the surgery in 45–90 min resulted in fewer complications (16%; 4/25) than completing it in more than 90 min (71%; 5/7; *p* = 0.01). No significant effect of age (*p* > 0.11), breed (*p* > 0.07), duration of dystocia (*p* = 1), and global experience (*p* > 0.12) was observed on mares’ intraoperative complication rate.

Sixty-five percent (22/34) of mares surviving surgery developed at least one postoperative complication.

These included 12 wound complications (5 dehiscences and 7 abscesses of the muscle wall), 5 endotoxic shock, 4 retained placentas, 3 peritonitis as well as one metritis, one muscle wall hematoma, one laminitis and one depression with decreased appetite. Four mares developed two complications, and one mare had three complications. All the surgical procedures of less than 45 min or more than 90 min led to postoperative complications. Out of the 24 surviving mares in the intermediate group (45-90 min), 50% suffered postoperative complications. Performing the surgery in 45–90 min resulted in fewer postoperative complications than completing it in more than 90 min (*p* = 0.019). No significant effect of age (*p* > 0.21), breed (*p* > 0.5), duration of dystocia (*p* > 0.09), global experience (*p* > 0.6), type of practice (*p* > 0.27), and experience in bovine C-section of the practitioner (*p* = 0.21) was observed on mares’ postoperative complication rate.

Veterinarians with the most experience (>20 years of practice) had fewer wound complications (8%; 1/12) compared to the intermediate 6–10 years group (55%; 6/11; *p* = 0.027) and 11–20 (67%; 4/6; *p* = 0.022). The use of a multifilament for the muscle wall (48%; 10/21 versus 0%; 0/10 for monofilament; *p* = 0.012), and an absorbable suture for the skin (67%; 6/9 versus 25%; 6/24 for non-absorbable, *p* = 0.044) was associated with significantly more wound complications. Using an epidural was also associated with fewer wound complications (0%; 0/9 versus 50%; 12/24; *p* = 0.009). Neither the side of surgery (*p* = 1) nor the number of sutured planes for muscle closure (*p* > 0.15) was associated with the wound complication rate. Similarly, the choice of the antiseptic agent for the surgical site preparation (*p* = 0.069), the pattern of local anesthesia (*p* = 0.35), the suture pattern (p = 1 and *p* > 0.135 for the muscle and the skin respectively), the choice of antibiotics (*p* > 0.2), and the type of wound protection (*p* > 0.057) were not associated with the wound complication rate.

The perioperative survival rate of the mares within 48 h was 91% (32/35). Besides a mare that died from a uterine hemorrhage during the surgery, the two remaining mares died from peritonitis and/or endotoxic shock. Thereafter, five mares also died from peritonitis and/or endotoxic shock between 48 h and 10 days postoperatively. One more died within a month after surgery (for an unknown reason). One mare was still alive after 3 months but her status at 1 year was unknown. All the remaining mares were alive at 1 year (74%; 25/34). The presence of an intraoperative or postoperative complication decreased the survival rate at 1 year (91%; 22/24 versus 30%; 3/10; *p* = 0.0006, and 100%; 12/12 versus 62%; 13/21; *p* = 0.03, respectively). The survival rate at 1 year was also negatively impacted (*p* = 0.0056) when surgery lasted more than 90 min (29%; 2/7) compared with the 45–90 min group (88%; 21/24). No significant effect of age (*p* = 0.16), breed (*p* > 0.46), duration of dystocia (*p* = 1), global experience (*p* > 0.55), type of practice (p = 1), and experience in bovine C-section of the practitioner (*p* > 0.23) was observed on mare survival rate.

Nine foals were still alive when the surgery was initiated (26%). Of these, all were delivered alive, but one died a few minutes after the birth due to prematurity and asphyxia. Another one died within a month for an unreported/unknown reason. The seven foals still alive after 1 month (78% of foals delivered alive – 20% of all cases) survived longer than 6 months (Table 4). Mares in the groups <6-year-old delivered significantly more live foals than mares in the 11 to 16-year-old group (63%; 5/8 versus 0%; 0/10; *p* = 0.0065). More live foals were delivered (*p* < 0.01) when dystocia lasted less than 2 h (Table 4). Veterinarians with more than 20 years of experience delivered significantly more live foals (50%; 6/12) than veterinarians with experience between 6 to 10 years (0%; 0/11; *p* = 0.0137).

**Table 4:** Foal survival rates according to the duration of the dystocia.

Duration of dystocia	Number of foals	Foals alive at the surgery and at delivery	Foals alive at 6 months
30 min-1 h	2	2 (100%)	2 (100%)
1-2 h	10	6 (60%)	4 (40%)
>2 h	23	1 (4%)	1 (4%)
Total	35	9/35 (26%)	7/35 (20%)
			7/9 (78%)

## 4 Discussion

In this study, most of the veterinarians had experience in bovine C-section, and generally worked with farm owners whose agricultural activity is governed by the economic context, leading to more opportunities to attempt standing C-sections on mares in the field. This is supported by the results of our survey where the economic aspect largely influences the decision to perform an in-field standing C-section (83% of cases). In this economic context, the decision to attempt a standing C-section in the field was mainly driven by the desire to save the mare as a priority (66%).

Paradoxically, foals’ survival concern was only mentioned in 9% of cases. This may be partially explained by the fact that only 26% of foals were still alive before surgery, which in turn questions the motivation to perform a C-section instead of a fetotomy. Most horses in this survey were draft-breed horses (40%) and Shetlands ponies (21%), which is consistent with the literature reporting that these breeds are predisposed to dystocia, due to their tendency to produce too large foals for the maternal pelvis (16). On the other hand, given the extreme sizes of these breeds, obstetric manipulations may be complicated, either because of difficult manipulation of the foal due to its large size and weight or to limited access through a small size pelvic passage. This observation could partly explain the choice of C-section rather than fetotomy in small size breeds (6). In addition, any prolonged and/or inappropriate vaginal manipulations, including fetotomy, could jeopardize the fertility of the mare (4, 17, 18).

The standing C-section could avoid the many disadvantages of recumbency and general anesthesia which can be risky on a shocked or debilitated horse. It then enables to minimize the side effects such as hypotension, arrhythmia, hypoxemia and hypercapnia, potentially improving the foal’s survival chances (19). In our survey, different sedation protocols have been described, most of them combining an alpha-2-agonist and an opioid with local anesthesia, which is coherent with the literature (13–15). Epidural was performed in 9 mares (26%), including 4 cases with foals still alive before C-section. According to some authors, performing an epidural before a C-section is often too time-consuming to allow fetal survival (20). Nevertheless, if the epidural was previously carried out in an attempt at assisted delivery, it would not delay the surgery. The beneficial association of the epidural on the wound complication rate may be related to easier manipulation of the uterus and abdominal wall relaxation. Another explanation is that more experienced bovine veterinarians might be more prone to use an epidural. If a direct beneficial effect should be confirmed, the decision to perform an epidural should balance time delays affecting fetal viability with beneficial effects on abdominal wound closure.

In this survey, 43% of mares were operated on the right flank and 57% on the left flank, without any association between surgical access side and wound complication rate. It has been reported that access via the right flank may reduce the risk of intestinal hernia through the wound and it was preferred in other reported standing C-sections (13–15). Based on our results, either side of the mare seems suitable for C-section and this choice depends on the situation and the surgeon’s preference.

The uterus was closed in 2 layers in 94% of cases. The sutures were performed using a monofilament in most mares (77%), likely because the use of this material is known to reduce the risk of infection by capillarity (21). A hemostatic suture was performed in 23% of the mares. The two mares whose uterus was sewn up in one layer also had a hemostatic suture. According to Vandeplassche et al. (9), the hemostatic suture prevents uterine bleeding induced by the specific equine diffuse placentation, but its use remains controversial. Some authors think that the expected benefit does not justify the added time, whereas others think that once the foal is delivered, there is less emergency, justifying taking time for this suture that could avoid complications (1, 4, 10, 22, 23). Compression and ligation only of the biggest uterine vessels followed by an invaginating suture has been suggested as an adequate alternative to prevent severe hemorrhage (6). In this study, the only mare that developed a fatal hemorrhage was one out of the 19 that had no hemostatic suture. However, the bleeding occurred after the detorsion of the uterus, and it was not clear if a suture could have modified the outcome.

In this report, 66% of mares who survived the C-section developed postoperative complications. These results do not seem to differ from the 72% obtained in the literature during C-sections under general anesthesia (2). During this study, no link has been established between the rate of postoperative complications and age, breed, and duration of dystocia. However, a previous study has shown that the longer the duration of dystocia, the higher the rate of complications during C-sections (2). It can be proposed that lengthened dystocia duration leads to increase the bacterial load of the uterus and the risk of contamination of the abdominal wound during the hysterotomy. The prolonged exposure of the abdominal cavity to a non-sterile environment in the field might also explain the relationship between the increased surgery time and higher postoperative complication rates. Alternatively, or additionally, the increased surgery time might reflect a more complicated surgery. Nonetheless, the connection between the surgery time and the complication rates makes unsurprising the negative association of all these factors on the survival rate of the mares. Interestingly but not surprisingly, surgeries performed by experienced rural practitioners who have developed specific skills in bovine C-sections seem associated with a reduced risk of complications.

During a standing C-section, the risk of wound dehiscence and seroma may be higher (14). Accordingly, wound dehiscence and/or muscle wall abscesses are the major complications encountered in this study, affecting a third of the mares. These complications can usually be managed by implementing local care for a second intention healing (14). A recent paper about standing laparotomy for managing equine colics reports a similar rate (30%) of surgical site infection in the 20 surviving mares (24), while this rate ranges from 0 to 30% for both emergency and elective recumbent C-sections in other reports (1, 2, 5, 7, 8, 10, 12). Surprisingly, the surgeons’ personal protective equipment did not influence the wound complication rate. It can be assumed that wearing a fully sterile suit in a field environment is less important than cleanliness and speed of action, especially for clean-contaminated surgeries like C-sections. In our study, the use of a multifilament material for muscle wall closure is significantly associated with wound complications. The same observation has already been made for the *linea alba* when using polyglactin for colic surgery and the use of a monofilament such as polydioxanone was then suggested (25). More surprisingly, in our survey, using absorbable material for skin closure was significantly and negatively associated with the wound complication rate. A potential explanation may reside in a chronic foreign body effect with infection by capillarity if sutures are left in place because of their absorbable nature.

For wound protection, an antiseptic spray was applied on half of the mares. Together with the use of Ford interlocking sutures for skin closure, which is common practice for C-sections in cattle (26), this routine likely reflects the habits of bovine practitioners. The literature recommends the placement of an abdominal bandage to reduce the prevalence of wound complications after laparotomy under general anesthesia in horses (27). In our study, no association between the wound complication rate and the type of protection applied was established, but only one mare had an abdominal bandage. Standing surgery avoids recovery from anesthesia, which can prevent wound contamination or stretching during recovery. Additionally, horses spend less time daily lying down than cattle. Together, these characteristics can protect the wound and make it less important to cover.

In this survey, the placenta was removed during surgery in 16 mares. Among the 18 remaining mares, only 4 (22%) had a retained placenta, which compares favorably with the literature that recognized retained placenta as one of the most frequent complications of C-section (1, 2, 5–8).

The mare survival rate for standing C-section in the field reported in our survey, which compares to previously reported data (13–15), as well as the foal survival rate we observed do not differ from rates described in the literature for emergency in-hospital procedures performed in recumbency (1, 5, 7, 9, 11, 12). The Table 5 summarizes some information about the specific conditions and outcomes of C-sections in recumbency.

**Table 5:** Summary and specificity of articles dealing with recumbent C-section in mares.

Reference	Number/Nature of C-sections	Comments/Survival rates
Vandeplassche et al. (9)	63 C-sections including 2 electives	- Authors report “most of them in recumbency”
		- General anesthesia
		- Low flank incision preferred to median or paramedian approach
		- Unspecified number of standing C-sections
		- High flank incision
		- Mare survival: 81%
		- Foal survival: 29%
Edwards et al. (8)	14 elective C-sections on 12 mares	- Production of gnotobiotic foal
		- Pony mares
		- General anesthesia
		- Low Flank approach
		- Mare survival: 92%
		- Foal survival: 78%
Vandeplassche, (9)	77 C-sections	- Surgical method and approach unspecified
		- Considered to be the actualized values from Vandeplassche et al. (9)
		- See comments above
		- Mare survival: 81%
		- Foal survival: 30%
Watkins et al. (10)	8 elective C-sections on 5 mares	- General anesthesia (half of cases only Guaifenesin until delivery of the foal)
		- Ventral midline celiotomy
		- Mare survival: 100%
		- Foal survival: 87%
Juzwiak et al. (5)	19 C-sections including 2 electives	- General anesthesia
		- Ventral midline celiotomy
		- Mare survival: 89%
		- Foal survival: 11%
Freeman et al. (7)	116 mares with C-section or vaginal delivery	- General anesthesia
	66 sections including 10 elective and 8 during colic surgery	- Ventral midline celiotomy
		- Mare survival: 82% (for all C-sections)
		- Foal survival: 14% (for all methods of delivery; not specifically C-section)
Byron et al. (11)	247 mares with dystocia including 61 C-sections	- General anesthesia
		- Ventral midline celiotomy
		- Mare survival: 89%
		- Foal survival: 26%
Maaskant et al. (12)	45 C-sections for dystocia	- General anesthesia
		- Ventral midline celiotomy
		- Mare survival: 91%
		- Foal survival: 31%
Abernathy-Young et al. (2)	95 C-sections including 71 dystocias (15 with prior fetotomy), 4 electives and 20 cases for concurrent mares’ disease	- General anesthesia
		- Ventral midline celiotomy
		- Mare survival: 84%
		- Foal survival: 35%
Gandini et al. (6)	7 C-sections for dystocia					- Ponies and Shetland	- Low flank approach in recumbency under sedation and local anesthesia	- In-field surgeries	- Mare survival: 100%	- Foal survival: 0%

Our data confirms that the duration of dystocia does not affect the outcome for the mare but seems decisive for the foal’s survival both in our study and the literature (1, 2, 7, 9, 11, 12). This finding certainly correlates with the rapid separation of the placenta, and impingement of the umbilical cord and the thorax in the pelvic canal when dystocia occurs, leading to foal asphyxia.

## 5 Limits of the study

The limitations of a survey-based retrospective study should be considered such as a low general response rate associated with this infrequent surgery and missing and/or imprecise responses as well as a bias consisting of reporting positive experiences more easily. In addition, criteria for diagnosing complications were left to the practitioners, leaving a place for personal interpretation in characterizing certain of them. Consequently, due to the limited number of cases as well as the nature of the study, statistical results on risk factors should be considered with caution. Also, the questionnaire did not cover all the aspects of the procedure, potentially missing relevant information, especially details on physical restraint, surgical access, and manipulation of the uterus at the opening. Finally, the conversion of some continuous data into categorical data (age of the mare, experience of the veterinarians in years or in performing C-sections in cows) constructs arbitrary groups.

## 6 Conclusion

Although the standing C-section in the field is rarely performed, it should not necessarily be considered a salvage or hopeless procedure. According to our survey, it appears to be a viable technique both for the survival of the dam and the foal and from an economic point of view. The candidates would mainly be mares that are known to be predisposed to dystocia, like heavy breeds or Shetlands and young mares, because their size does not always allow obstetrical manipulations, and because their economic value is low while their emotional value may be quite high. A prospective study on a larger population to confirm these results and to determine the impact of the standing C-section on the future fertility of the mare would be useful.

## Data Availability

The datasets presented in this article are not readily available due to confidentiality for veterinarians having fulfilled the questionnaire. Requests to access the datasets should be directed to g.delarebieredepouyade@uliege.be.
