# Supplementary material for: Survey on outcomes of emergency standing caesarean section in equids
Source: Front Vet Sci. 2025 Apr 24;12:1548978. doi: 10.3389/fvets.2025.1548978 (PMC12061027; doi:10.3389/fvets.2025.1548978)
Supplement: Supplementary file 1 [file Table_1.docx]

**Annex 1: Questionnaire: The in-field standing caesarean section in the mare**

**n = The number of answers for each question.**

- 1. **Veterinarian informations**

1. Your name (or pseudonym if you wish to remain anonymous)

n=35

1. How many years of experience do you have ?

n=35

1. In what country did you practice at the time of the intervention ?

n=35

1. What is your type of practice?

n=35

*Only one possible answer.*

- Rural
- Equine
- Mixed (Rural- Equine)
- Mixed (Small animal -Equine)
- Others:

1. If you practice rural medicine, how many cesarean sections have you already performed on cows?

n=35

*Only one possible answer.*

- < 50
- 50-100
- >100

1. How many standing cesarean sections have you already performed on the mare?

n=35

- 1. **Mares’ information**

1. What is the breed of the mare?

n=35

1. How old was the mare at the time of surgery?

n=32

1. Was the mare primiparous?

n=35

*Only one possible answer.*

- Yes
- No

10. If the mare was pluriparous, how many gestation had she before?

n =15

1. If the mare was pluriparous, has she presented complications during her previous pregnancies/ foalings?

n =17

*Only one possible answer*

- Yes
- No

1. What was the reason that led you to make the decision to perform a standing cesarean section?

n=35

*Several possible answers*

- The clinic was too far away
- The horse was not transportable
- The financial aspect for the owner
- Vital emergency to save the mare
- Vital emergency to save the foal
- Others:
  1. **Course of the cesarean section**

# How long had the work started when the intervention began?

n=35

*Only one possible answer*

- < 30 minutes
- 30 minutes - 1hour
- 1h - 2h
- >2h

# How long did the procedure take (from asepsis to closure)?

n=35

*Only one possible answer*

- < 45 minutes
- 45 minutes - 1h30
- > 1h30

# Was the foal alive before the onset of the cesarean section ?

n=35

*Only one possible answer*

- Yes
- No

# Did the mare lie down during the operation ?

n=35

*Only one possible answer*

- Yes
- No

1. Did you have any complications during the procedure?

n=35

*Only one possible answer*

- Yes
- No

1. If you had any complications, what were they?

n =10

*Several possible answers*

- Excessive uterine bleeding
- Excessive bleeding from the abdominal wall
- Uterine muscle tear
- Parietal muscle tear
- Abdominal contamination by fetal fluids
- Others:
  1. **Protocol of the intervention**

***Animal asepsis protocol***

1. Preparation of the animal

n=35

*Several possible answers*

- Clipping
- Shaving
- Others:

1. Asepsis of the animal

n=35

*Several possible answers*

- Chlorhexidine
- Povidone iodine
- Others:

# Rinsing

n=35

*Several possible answers*

- Alcohol
- Water
- Others:

# ***Surgeon's asepsis protocol***

1. Hands asepsis

n=35

*Only one possible answer*

- Chlorhexidine
- Povidone iodine
- Hydroalcoholic gel
- Others:

# Hands rinsing

n=35

*Only one possible answer*

- Alcohol
- Water
- Nothing
- Others:

1. Surgeons’ Equipment

n=35

*Several possible answers*

- Sterile gloves
- Non-sterile gloves
- Sterile gown
- Non-sterile gown
- Mask
- Others:

***Surgery protocol***

1. What analgesia/sedation protocol did you use to perform your procedure?

n=34

*Several possible answers*

- acepromazine
- a2- agonist IV
- a2-agonist IM
- butorphanol IV
- butorphanol lM
- ketamine IV
- ketamine IM
- morphine IV
- morphine IM
- caudal epidural
- thoracolumbar paraspinal blocks
- local anesthesia "line block"
- local anesthesia "L'.' inversed (7-block)
- Others:

1. Did you administer any clenbuterol during the procedure?

n=35

*Only one possible answer*

- Yes
- No

# If yes, how many times did you administer it?

n =17

1. What was the access to the surgery?

n=35

*Only one possible answer*

- From the right flank
- From the left flank

# ***Suture of the uterus***

1. Did you completely remove the placenta before the uterus closed?

n=34

*Only one possible answer*

- Yes
- No

# If you left the placenta, did you peel it off the sides of the uterine wound margins?

n =18

*Only one possible answer*

- Yes
- No

# Did you perfom an hemostatic continuous suture on the uterine wound margins?

n=35

*Only one possible answer*

- Yes
- No

1. How many layers of suture did you perform? (Not including hemostatic suture)

n=35

*Only one possible answer*

- 1 layer
- 2 layers
- Others:

1. Which type of suture pattern did you use?

n=35

*Several possible answers*

- Invaginating continuous
- Simple continuous
- Other:

1. Which type of thread did you use?

n=35

*Several possible answers*

- Absorbable
- non-absorbable
- monofilament
- multifilament

***Suture of muscles***

1. In how many plans did you suture the muscle wall?

n=31

*Only one possible answer*

- 2 (Transverse muscle then internal oblique and external oblique muscles)
- 2 (Transverse muscle and internal oblique muscle then external oblique muscles)
- 3 (Transverse muscle - internal oblique muscle - external oblique muscles)
- Others

1. Which type of suture pattern did you use?

n=35

*Only one possible answer*

- simple continous
- interrupted (simple, cruciate "X", mattress "U")
- Others

1. Which type of thread did you use?

*Several possible answers*

- absorbable
- non-absorbable

n=35

- monofilament
- multifilament

n=32

***Suture of the skin***

# Which type of suture pattern did you perform?

n=35

*Only one possible answer*

- Ford interlocking continuous
- simple continuous
- simple interrupted
- cruciate "X" interrupted
- horizontal or vertical mattress ("U") interrupted
- Others

# Which type of thread did you use?

*Several possible answers*

- Absorbable
- Non-absorbable

n=35

- monofilament
- multifilament

n=32

# ***Post-operative care***

1. What type of wound protection did you use?

n=34

*Several possible answers*

- None: wound left uncovered
- surgical dressing
- abdominal bandage
- antibiotic spray
- antiseptic spray
- Others:

# Did you administer antibiotics?

n=35

*Only one possible answer*

- Yes
- No

# If yes, which family(ies) of antibiotics did you administer?

n=35

*Several possible answers*

- penicillins
- cephalosporins
- aminosides
- sulfonamides
- tetracyclines
- macrolides
- fluoroquinolones
- Others:

# If yes, for how long did you administer it?

n=32

1. Did you administer anti-inflammatory?

n=35

*Only one possible answer*

- Yes
- No

1. If yes, which type of anti-inflammatory did you administer?

n=34

*Only one possible answer*

- flunixin meglumine
- meloxicam
- phenylbutazone
- ketoprofen
- carprofen
- firocoxib
- steroidal anti-inflammatory
- Others:

1. If yes, for how long did you administer it?

n=29

- 1. **After the surgery**

1. If you did not remove the placenta during the procedure, did you administer any oxytocin?

n=18

*Only one possible answer*

- Yes
- No

1. If yes, which route of administration did you use to give the oxytocin ?

n=14

*Only one possible answer*

- intra-muscular
- intravenous

1. How long after the end of the procedure did you administer the oxytocin?

n=14

1. If you did not remove the placenta during the procedure, how long afterwards did the mare expel it?

n=16

*Only one possible answer*

- < 1h
- 1h-3h
- 4h-6h
- > 6h

1. Did you have any complications on the mare in the next hours/days/months after the procedure ?

n=34

*Only one possible answer*

- Yes
- No

1. If you had any complications, which were they?

n=22

*Several possible answers*

- wound dehiscence
- peritonitis
- uterine hemorrhage
- placental retention
- metritis
- laminitis
- endotoxinic shock
- wall abscess
- others:

1. Did the mare survive after the procedure ?

n=35

*Only one possible answer*

- Yes
- No

1. If no, which was the cause of the death?

n=9

*Several possible answers*

- Peritonitis
- uterine hemorrhage
- Endotoxin shock
- Unknown
- others:

1. How long did the mare survive?

n=35

*Only one possible answer*

- < 10 days
- 10 days – 1 month
- 1 month – 6 months
- 6 months - 1 year
- > 1 year

1. Was the foal alive after the surgery?

n=35

*Only one possible answer*

- Yes
- No

1. If yes, did the foal develop any problems in the next hours/days/weeks/months after the procedure?

n=9

*Only one possible answer*

- Yes
- No

1. If yes, which complication(s) did it present?

n=2

*Several possible answers*

- weakness
- septicemia
- colic
- omphalitis
- arthritis
- others:

1. Did it need hospitalization due to complications?

n=2

*Only one possible answer*

- Yes
- No

1. How long did the foal survive?

n=9

*Only one possible answer*

- < 1 week
- 1 week - 1 month
- 1-3 months
- 3 - 6 months
- > 6 months

1. If the foal did not survive, which was the cause of the death?

n=2

*Several possible answers*

- neonatal maladjustment/ prematurity/ asphyxia
- septicemia without signs of prematurity
- colic
- unknown
- others:
  1. **Return to reproduction**

1. Has the mare been returned to breeding ?

n=24

*Only one possible answer*

- Yes
- No

1. If yes, how long afterwards?

n=5

1. If yes, how many cycles were necessary to pregnancy?

n=2

*Only one possible answer*

- 1 cycle exploited
- 2 cycles exploited
- ≤3 cycles exploited

1. Has the mare suffered from any problems during her pregnancy ?

n= 4

*Only one possible answer*

- Yes
- No

1. If yes, which problem(s) did she encounter?

n= 1

*Only one possible answer*

- Placententis
- Abortion
- Dystocia
- Others :

1. Did the next parturition require the intervention of a veterinarian?

n=4

*Only one possible answer*

- Yes
- No

1. If yes, which type of intervention was needed ?

n=2

*Only one possible answer*

- Assisted vaginal delivery
- Controlled vaginal delivery (general anesthesia - hind legs raised)
- Standing caesarean section
- Caesarean section in recumbency
- Others:
